# Fast Recognition and Counting Method of Dragon Fruit Flowers and Fruits Based on Video Stream

**DOI:** 10.3390/s23208444

**Published:** 2023-10-13

**Authors:** Xiuhua Li, Xiang Wang, Pauline Ong, Zeren Yi, Lu Ding, Chao Han

**Affiliations:** 1 School of Electrical Engineering, Guangxi University, Nanning 530004, China; lixh@gxu.edu.cn (X.L.); 2112391054@st.gxu.edu.cn (X.W.); dinglu@gxu.edu.cn (L.D.); hanchao@gxu.edu.cn (C.H.); 2 Guangxi Key Laboratory of Sugarcane Biology, Guangxi University, Nanning 530004, China; 3 Faculty of Mechanical and Manufacturing Engineering, Universiti Tun Hussein Onn Malaysia, Parit Raja 86400, Johor, Malaysia; ongp@uthm.edu.my

**Keywords:** YOLOv5, ByteTrack, fruit detection, object detection, object counting

## Abstract

Dragon fruit (*Hylocereus undatus*) is a tropical and subtropical fruit that undergoes multiple ripening cycles throughout the year. Accurate monitoring of the flower and fruit quantities at various stages is crucial for growers to estimate yields, plan orders, and implement effective management strategies. However, traditional manual counting methods are labor-intensive and inefficient. Deep learning techniques have proven effective for object recognition tasks but limited research has been conducted on dragon fruit due to its unique stem morphology and the coexistence of flowers and fruits. Additionally, the challenge lies in developing a lightweight recognition and tracking model that can be seamlessly integrated into mobile platforms, enabling on-site quantity counting. In this study, a video stream inspection method was proposed to classify and count dragon fruit flowers, immature fruits (green fruits), and mature fruits (red fruits) in a dragon fruit plantation. The approach involves three key steps: (1) utilizing the YOLOv5 network for the identification of different dragon fruit categories, (2) employing the improved ByteTrack object tracking algorithm to assign unique IDs to each target and track their movement, and (3) defining a region of interest area for precise classification and counting of dragon fruit across categories. Experimental results demonstrate recognition accuracies of 94.1%, 94.8%, and 96.1% for dragon fruit flowers, green fruits, and red fruits, respectively, with an overall average recognition accuracy of 95.0%. Furthermore, the counting accuracy for each category is measured at 97.68%, 93.97%, and 91.89%, respectively. The proposed method achieves a counting speed of 56 frames per second on a 1080ti GPU. The findings establish the efficacy and practicality of this method for accurate counting of dragon fruit or other fruit varieties.

## 1. Introduction

Dragon fruit (*Hylocereus undatus*), also known as pitahaya or pitaya, is a tropical and subtropical fruit that has gained popularity among consumers due to its nutritional value and pleasant taste [1,2]. China stands as the leading global producer, with an annual output of 1.297 million tons in 2019. Accurate monitoring of flower and fruit quantities during various stages is vital for growers to forecast yield and optimize order planning. Nevertheless, the prevalent reliance on labor-intensive, time-consuming, and inefficient manual counting methods persists among dragon fruit growers in different regions [3,4].

With the advancement of information technology, machine vision technology has emerged as a significant tool for plant monitoring and yield estimation. Its benefits, including cost-effectiveness, ease of acquisition, and high accuracy, have propelled its adoption. Orchards typically feature wide row spacing, enabling the collection of video data using ground moving platforms, facilitating large-scale detection. Video-based target statistics involve two essential steps: target recognition and target tracking. Traditional target recognition algorithms such as the Deformable Part Model (DPM) [5] and Support Vector Machine (SVM) have been gradually replaced by deep learning algorithms due to their limitations in detection accuracy, robustness, and speed.

Currently, deep learning-based object detection algorithms can be broadly categorized into two-stage detection algorithms based on candidate regions and one-stage detection algorithms based on regression principles. Two-stage detection algorithms, such as Faster R-CNN [6], have found extensive application in fruit (prickly pear fruit [7], apples [8], passion fruit [9], etc.) detection, crop (Sugarcane Seedlings [10], weeds [11]) detection, disease (sweet peppers disease [12], soybean leaf disease [13]) detection, and so on, achieving accuracy levels ranging from 55.5% to 93.67%, depending on the complexity and difficulty. However, those kinds of two-stage algorithms have obvious drawbacks of being large in size, slow in speed, and challenging to integrate into mobile devices.

One-stage detection algorithms, prominently represented by the YOLO [14,15,16] series, are preferred for real-time performance requirements owing to their compact size, rapid speed, and high accuracy. YOLO series models were built for variable objects in agriculture, such as detection of weeds [17] or diseases [18] with a similar appearance or features. YOLO models have also shown balanced performance on fruit detection though the biggest challenge that exists in the similar colors and occlusion in some conditions. Wang [19] employed YOLOv4 [20] for apple detection, achieving an average detection accuracy of 92.23%, surpassing Faster R-CNN by 2.02% under comparable conditions. Yao [21] utilized YOLOv5 to identify defects in kiwi fruits, accomplishing a detection accuracy of 94.7% with a mere 0.1 s processing time per image. Yan [22] deployed YOLOv5 to automatically distinguish graspable and ungraspable apples in apple tree images for a picking robot. The method achieved an average recognition time of 0.015 s per image. YOLO models of different versions also have performance differences. Comparative analysis revealed that YOLOv5 enhanced the model’s mean Average Precision (mAP) by 14.95% and 4.74% compared to YOLOv3 and YOLOv4, respectively, while significantly compressing the model size by 94.6% and 94.8%. Moreover, YOLOv5 demonstrated average recognition speeds 1.13 and 3.53 times faster than YOLOv4 and YOLOv3 models, respectively. Thus, the YOLOv5 algorithm effectively balances speed and accuracy in one-stage detection algorithms.

Conventional target tracking methods, including optical flow [23] and frame difference [24], exhibit limited real-time performance, high complexity, and susceptibility to environmental factors. Conversely, tracking algorithms based on Kalman filtering [25] and Hungarian matching [26], such as SORT [27], DeepSORT [28], and ByteTrack [29], excel in rapid tracking speed and high accuracy, meeting the demands of video detection. SORT optimizes tracking efficiency by correlating frames before and after an image, yet struggles to address occluded target tracking challenges. DeepSORT enhances occluded target tracking by leveraging deep appearance features extracted through a convolutional neural network (CNN), augmenting the deep feature matching capability, albeit at the expense of detection speed. Similar to SORT, ByteTrack does not employ deep appearance features but solely relies on the inter-frame information correlation. However, ByteTrack effectively addresses occluded target recognition challenges by emphasizing low-confidence detection targets, thereby achieving a good balance between tracking accuracy and speed. In summary, ByteTrack surpasses other tracking algorithms in performance, yet it is restricted to single-class target tracking and does not provide direct support for multi-class target classification tracking.

To address existing challenges of current tracking algorithms and meet practical demands, this study introduces an enhanced ByteTrack tracking algorithm for real-time recognition, tracking, and counting of dragon fruit at various growth stages in inspection videos. The proposed method comprises three core modules: (1) a dragon fruit multi-classification recognition model based on YOLOv5; (2) the multi-class detection outcomes are utilized as inputs for the improved ByteTrack tracker, enabling end-to-end tracking of dragon fruit across different growth stages; (3) regions of interest (ROI) are defined to accomplish classification statistics for dragon fruit at distinct growth stages. This method can be further integrated into mobile devices, facilitating automated inspection of dragon fruit orchards and offering a viable approach for timely prediction of dragon fruit yield and maturity.

The organization of the rest of this paper is as follows. In Section 2, we present the proposed method and performance metrics in detail. Section 3 is dedicated to the discussion of our results. Following that, in Section 4, we delve into the discussion of future work. Finally, in Section 5, we draw our conclusions.

Our contributions include (1) an enhanced ByteTrack tracking algorithm was proposed to simultaneously recognize, track, and count dragon fruit of different ripeness at both sides; (2) the YOLOv5 object detector was employed as the detection component of the ByteTrack tracker, and multi-class tags were introduced into ByteTrack, achieving efficient and rapid tracking of multiple classes.

## 2. Materials and Methods

### 2.1. Image Acquisition for Dragon Fruit

**Figure 1:**
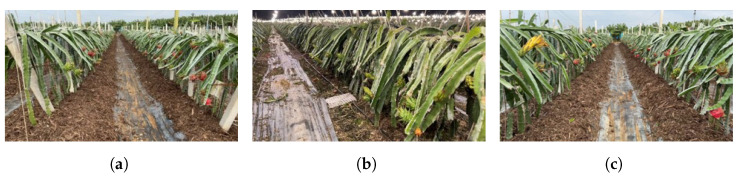
The plots with different scenarios: (**a**) plots with coexisting green and red fruits; (**b**) plots with coexisting dragon fruit flowers and green fruits; and (**c**) plots with coexisting flowers and green and red fruits.

A series of videos were recorded using several handheld smartphones along the inter-row paths of a dragon fruit plantation situated in Long’an, Guangxi, on 7 November 2021. Based on the observed conditions, the plots can be categorized into three scenarios: (a) plots with coexisting green fruits (immature dragon fruit) and red fruits (mature dragon fruit), as depicted in Figure 1a; (b) plots with coexisting dragon fruit flowers and green fruits, as shown in Figure 1b; and (c) plots with coexisting flowers, green fruits, and red fruits, as illustrated in Figure 1c. The video recordings were conducted in the afternoon and night time (3:00 p.m.–9:00 p.m.), with supplementary illumination provided by grow lights during night time. The smartphones were handheld with a gimble system at a height of 1.0–1.5 m above the ground. The camera lens was put straight forward, assuring the dragon fruit plants on both sides of the path being recorded. The lighting conditions included front light and backlight during the day. The video acquisition speed was about 1 m/s. The collected videos were stored in MP4 format, featuring a resolution of 1920 (horizontal) × 1080 (vertical) and a frame rate of 30 frames per second. In total, 61 videos were acquired, with a combined duration of approximately 150 min.

### 2.2. The Proposed Technical Framework

**Figure 2:**
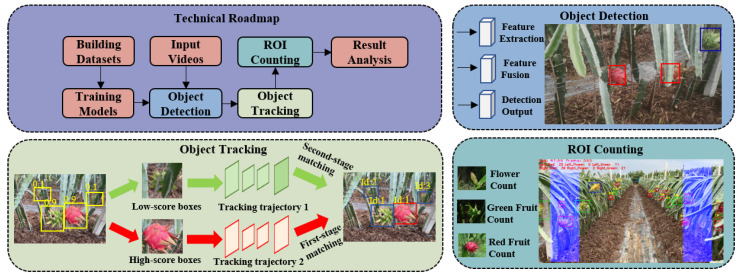
The proposed technical framework in this study.

The technical framework of this study, as illustrated in Figure 2, encompasses the following five key components:

(1) Construction of a dragon fruit detection dataset: Curating a dataset by selecting videos captured under diverse environmental conditions, extracting relevant key frames, and annotating them to establish a comprehensive dragon fruit detection dataset.

(2) Training a dragon fruit detection model: Utilizing the constructed dataset to train a detection algorithm, thereby developing a dragon fruit recognition model capable of identifying dragon fruit at distinct growth stages. The recognition outcomes serve as an input for subsequent object tracking processes.

(3) Tracking dragon fruit at different growth stages: Adding multi-classification information on the basis of multi-object tracking technology and integrating the results of dragon fruit detection to achieve real-time tracking of dragon fruit across various growth stages.

(4) Counting dragon fruit using the ROI region: Incorporating a region of interest (ROI) within the video and utilizing the results obtained from object tracking to perform accurate counting of dragon fruit at different growth stages within the defined ROI.

(5) Result analysis: Conducting a comprehensive assessment of the dragon fruit counting method by evaluating the performance of object detection, object tracking, and the effectiveness of counting within the ROI region.

### 2.3. Construction of the Dragon Fruit Dataset

Twenty videos, each with a duration ranging from 30 s to 3 min, were randomly selected for the study. Out of these, 16 videos were utilized to extract a raw image dataset by capturing one frame for every 30 frames. Images depicting figs or suffering from blurriness were manually eliminated. The resulting dataset comprised 5500 images, which were subsequently numbered for manual classification of flowers, green fruits, and red fruits using labelimg software. Targets exhibiting occlusion areas exceeding 90% or those with blurry attributes were excluded from the labeling process. Finally, the annotated dataset was divided into a training set (5000 images) and a validation set (500 images), maintaining a 9:1 ratio. The remaining four videos were employed for system testing purposes, encompassing frame rates spanning from 1800 to 5400 frames. Videos 1 and 2 were captured during night time, while videos 3 and 4 were obtained in daylight conditions, employing the same filming techniques involving the simultaneous recording of two rows of dragon fruit plants. The basic information of the dataset is listed in Table 1.

### 2.4. Multi-Object Tracking with Multi-Class

The ByteTrack algorithm, comprising object detection and object tracking stages, encounters challenges regarding real-time performance in detection and the lack of class information in tracking. To address these limitations, this study presents an enhanced version of the algorithm for efficient detection and tracking of dragon fruits at various growth stages. Notably, we replaced the original detector in ByteTrack with YOLOv5 to enhance both accuracy and speed of detection. Additionally, multi-class information was integrated into the tracking module so that the object numbers of specific classes could be counted.

#### 2.4.1. Object Detection

The YOLO (You Only Look Once) algorithm is a renowned one-stage object detection algorithm that identifies objects within an image in a single pass. Unlike region-based convolutional neural network (RCNN)-type algorithms that generate candidate box regions, YOLO directly produces bounding box coordinates and class probabilities of each bounding box through regression. This unique approach enables faster detection speeds, as it eliminates the need for multiple passes or region proposal steps.

**Figure 3:**
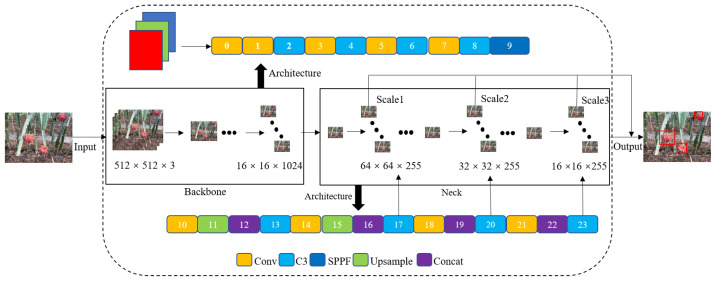
The network architecture of YOLOv5-s.

The YOLOv5 architecture offers four variants, distinguished by the network’s depth and width: YOLOv5-s, YOLOv5-m, YOLOv5-l, and YOLOv5-x. Among these, YOLOv5-s is the smallest model with the fastest inference speed, making it ideal for deployment on edge detection devices. In this study, we employed the YOLOv5-s network model, as depicted in Figure 3, which comprises four key components: Input, Backbone, Neck, and Output. The Input module performs essential pre-processing tasks on the dragon fruit images, including adaptive scaling and data augmentation using Mosaic. The Backbone module, consisting of C3 and SPPF structures, focuses on feature extraction from the input image. The C3 module divides the basic feature layer into two parts. One part conducts convolutional operations, while the other part fuses with the convolutional operation results of the first part through cross-layer combination to obtain the feature layer. The SPPF structure plays a crucial role in aggregating multi-scale features obtained from C3 to effectively expand the image’s receptive field. In the Neck module, the FPN+PAN structure is employed to fuse features extracted from different layers. FPN facilitates the propagation of features from top to bottom layers, while PAN enables the propagation of features from bottom to higher layers. By combining both approaches, features from diverse layers are fused to mitigate information loss. The Output module generates feature maps of various sizes. By analyzing these feature maps, the location, class, and confidence level of the dragon fruit can be predicted. In this study, 5000 images containing dragon fruit flowers and fruits were used as the training dataset. The information about the dataset was listed in Table 1.

#### 2.4.2. Object Tracking

Multiple Object Tracking (MOT) aims to estimate the bounding boxes and identify objects within video sequences. Many existing methods for MOT achieve this by associating bounding boxes with scores exceeding a predefined threshold to establish object identities. However, this approach poses a challenge when dealing with objects that have low detection scores, including occluded instances, as they are often discarded, thus resulting in the loss of object trajectories.

The ByteTrack algorithm introduces an innovative data association approach called BYTE, which offers a simple, efficient, and versatile solution. In contrast to conventional tracking methods that primarily focus on high-scoring bounding boxes, BYTE adopts a different strategy. It retains most of the bounding boxes and classifies them based on their confidence scores into high and low categories. Rather than discarding the low-confidence detection targets outright, BYTE leverages the similarity between the bounding boxes and existing tracking trajectories. This enables the algorithm to effectively distinguish foreground targets from the background, mitigating the risk of missed detections and enhancing the continuity of object trajectories.

**Figure 4:**
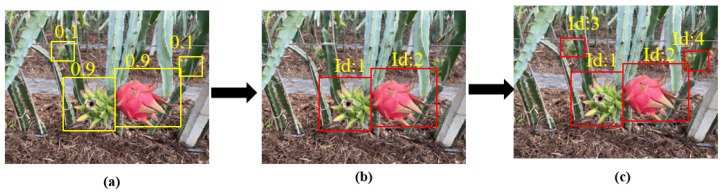
The workflow of BYTE. (**a**) Partitioning bounding boxes into high and low scoring categories; (**b**) matching of high-scoring boxes with existing tracking trajectories; and (**c**) matching of all bounding boxes.

The BYTE workflow, depicted in Figure 4, encompasses three key steps: (1) partitioning bounding boxes into high and low scoring categories; (2) prioritizing the matching of high-scoring boxes with existing tracking trajectories, resorting to low-scoring boxes only when the matching using high-scoring boxes is unsuccessful; (3) generating new tracking trajectories for high-scoring bounding boxes that fail to find suitable matches within existing trajectories, discarding them if no suitable match is found within 30 frames. By employing this streamlined data association approach, ByteTrack demonstrates exceptional performance in addressing the challenges of MOT. Moreover, its attention on low-scoring bounding boxes allows for effective handling of scenarios featuring significant object occlusion.

**Figure 5:**
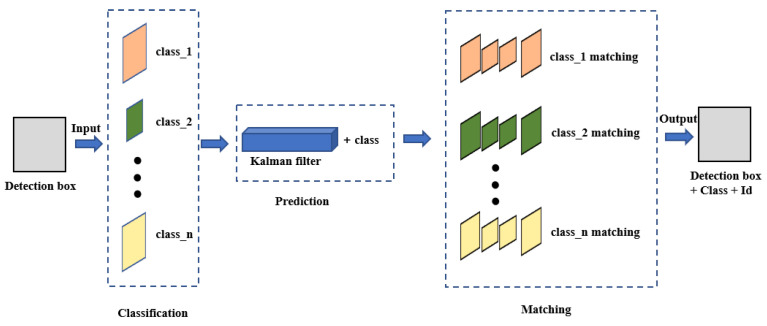
The tracking process of ByteTrack with multi-class information incorporated.

In this study, we incorporated multi-class information into the tracking process of ByteTrack, as depicted in Figure 5 with dashed boxes indicating the inclusion of multi-class information. Based on the category of the bounding box, a Kalman filter was utilized with classification information to enhance the prediction accuracy, as depicted in Equation (1), which represents the state equation.

(1) $$x={\left[u,v,s,r,\dot{u},\dot{v},\dot{s},class\right]}^{T}$$

In this equation, *u* and *v* denote the center point of the bounding box, *s* represents the aspect ratio, *r* corresponds to the height of the bounding box, $\dot{u}$, $\dot{v}$, and $\dot{s}$ denote their respective rates of change, and class indicates the category information of the bounding box. Following the generation of predicted objects, matching of tracking trajectories is performed based on the category. Upon successful matching, detection boxes with classification and identity IDs are outputted.

### 2.5. Counting Method Using the ROI Region

**Figure 6:**
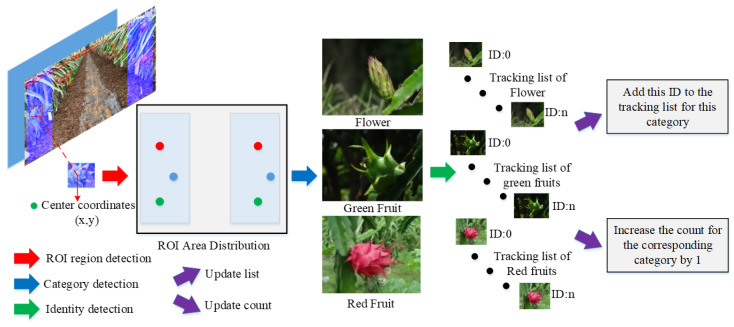
The workflow of the counting method using the ROI region.

Considering the characteristics of inspection videos, which involve single-side and double-side inspections, this paper presents a ROI counting method capable of counting on either one side or both sides. Here, we mainly focus on introducing the double-side ROI counting method, which enables simultaneous capture of both sides of the dragon fruit in the aisle. The layout of the double-side ROI region is shown in the inspection video schematic in Figure 6. In this schematic, a counting belt is positioned on each side of the video, highlighted by a blue translucent mask overlay. Additionally, the current video frame number, processing frequency, and statistics related to various categories of flowers and fruits are displayed in the upper-left corner of the video.

The counting method, as illustrated in Figure 6, involves two primary steps. (1) Frame-by-frame analysis: The center coordinates of the identified dragon fruit target box are assessed to determine if they fall within the designated ROI counting region. If they do not, the process is repeated. If the coordinates are within the ROI counting region, the procedure proceeds to the next step. (2) Categorization and tracking: The category of the flower/fruit target entering the ROI region is confirmed, and the corresponding category’s tracking list is checked for the presence of the target’s ID. If the ID is not already included in the tracking list, it is added, and the counter for the respective category is incremented by 1. If the ID is already present in the tracking list, it is not counted. Once all video frames have been analyzed, the tracking lists for all categories within the ROI region are cleared.

### 2.6. Evaluation Metrics

The Intersection over Union (IOU) is a widely used metric for evaluating the accuracy of object detection. It quantifies the overlap between the predicted bounding boxes and the ground truth boxes, as expressed in Equation (2). In this study, we employed an IOU threshold of 0.5, indicating that a detection is deemed correct if the IOU is greater than or equal to 0.5, and incorrect otherwise.

(2) $$I_{ou}=\frac{S_{A\cup B}}{S_{A\cap B}}$$

The intersection area between the predicted bounding box and the ground truth box is represented as $S_{A\cap B}$, while the union area is denoted as $S_{A\cup B}$. By considering the differences between the predicted and ground truth results, the samples can be categorized into four groups: true positive (TP), false positive (FP), false negative (FN), and true negative (TN). From these groups, various evaluation metrics can be derived, including accuracy (*P*), recall (*R*), average precision (AP), and mean average precision (mAP), which are expressed in Equations (3)–(6).

(3) $$P=\frac{TP}{TP+FP}$$

(4) $$R=\frac{TP}{TP+FN}$$

(5) $$AP=\int_{0}^{1}P\left(R\right)dR$$

(6) $$mAP=\frac{1}{3}\left(AP_{flower}+AP_{green\_fruit}+AP_{red\_fruit}\right)$$

The evaluation of the counting results is conducted using counting accuracy ($P_{c}$), counting precision ($AP_{c}$), and mean counting precision ($mAP_{c}$). These metrics are defined in Equations (7)–(9), where $N_{a}$ represents the automated counting values, $N_{t}$ represents the true values, and *N* denotes the total number of tested videos

(7) $$P_{c}=\left(1-\frac{\left|N_{a}-N_{t}\right|}{N_{t}}\right)\times 100\%$$

(8) $$AP_{c}=\frac{1}{N}\sum_{i=1}^{N}P_{c}\left(i\right)$$

(9) $$mAP_{c}=\frac{1}{3}\left(AP_{c_{flower}}+AP_{c_{green\_fruit}}+AP_{c_{red\_fruit}}\right)$$

### 2.7. Experimental Environment and Parameter Setting

In this study, the models were built and improved using the PyTorch deep learning framework. Model training and validation were conducted on Ubuntu 18.04. The computer used had an Intel(R) Core(TM) i7-8700K CPU of 3.70 GHz, 16 GB RAM, and an NVIDIA GeForce RTX 3090 GPU with 24 GB of memory. GPU acceleration was employed to expedite network training, utilizing Cuda version 11.3.0 and Cudnn version 8.2.0. Stochastic Gradient Descent (SGD) was chosen as the optimizer for neural network optimization and to accelerate the training process. The momentum parameter for SGD was set to 0.9, and the weight decay parameter was set to 0.0005. The input images were uniformly resized to 512 × 512 pixels, and a batch size of 16 was used during training.The trained model was then tested on another computer which has an NVIDIA GeForce GTX 1080ti GPU with 16 GB of memory.

## 3. Results

### 3.1. YOLOv5 Object Detection Results

**Figure 7:**
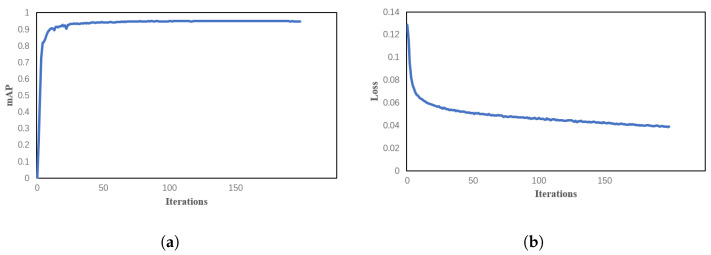
The curves of (**a**) loss; and (**b**) mAP during the training process.

The training progress of the YOLOv5 model for dragon fruit recognition is depicted in Figure 7, illustrating the curves for loss and mAP. Notably, the loss exhibits a rapid decline while mAP experiences a substantial increase throughout the training iterations. As training progresses, the loss value gradually converges towards the minimum of 0.04, reaching this threshold after approximately 150 iterations.

**Figure 8:**
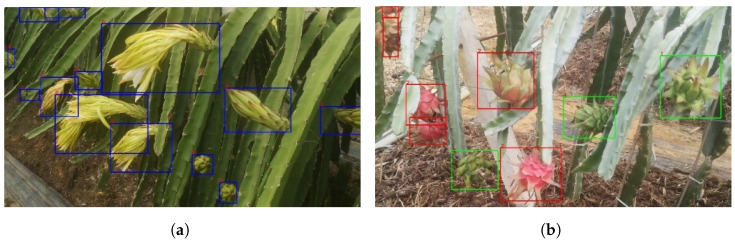
The recognition results obtained by the YOLOv5 model for dragon fruits in different growth stages. (**a**) Detection results of different types of dragon fruit flowers, and (**b**) detection results of red and green fruits.

Figure 8 presents the actual image recognition results obtained from the YOLOv5 model. The recognition outcomes are indicated by blue, green, and red boxes, corresponding to flowers, green fruits, and red fruits, respectively. In Figure 8a, the image highlights the significant variation in the size and shape of dragon fruit flowers due to factors such as blooming stage and shooting angle. Despite this challenge, the YOLOv5 detection model demonstrates the precise identification of the flowers. Figure 8b shows the recognition of green and red fruits in their coexisting state, where the detection model accurately distinguishes and identifies both types of fruits. This successful recognition serves as a crucial foundation for subsequent counting of dragon fruits across various growth stages.

Table 2 presents the recognition results of the YOLOv5 object detection model on the test set. The findings reveal that the red fruit achieves the highest recognition accuracy, reaching 96.1%. It is followed by the green fruit, which attains a recognition accuracy of 94.8%. The flowers exhibit the lowest recognition accuracy, yet still reach 94.1%. Overall, the average detection accuracy across the three categories is 95.0%. Furthermore, the model demonstrates a fast detection speed of 0.008s per frame image, equivalent to detecting 125 frames per second. These outcomes indicate the model’s capability to rapidly and accurately identify various flower and fruit targets, thereby providing essential technical support for subsequent tracking and counting tasks

### 3.2. Results of Object Tracking and Counting

**Figure 9:**
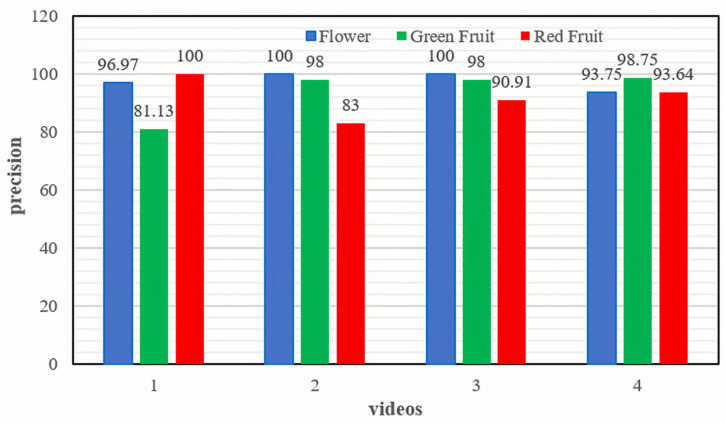
The counting accuracies achieved by the proposed counting method for videos collected at different locations and times.

To evaluate the performance of the counting method proposed in this study, four videos collected from different locations and times, as outlined in Table 3, were utilized. The obtained results, as illustrated in Figure 9, reveal notable insights. Notably, the counting accuracy for red fruits in Video 1 and flowers in Video 2 achieved a perfect score of 100%. However, the counting accuracy for green fruits in Video 1 and red fruits in Video 2 was comparatively lower, standing at 81.13% and 83%, respectively. Conversely, the counting accuracy for flowers in Video 3 and green fruits in Video 4 demonstrated the highest performance, reaching 100% and 98.75%, respectively. Nevertheless, the counting accuracy for red fruits in these videos was relatively lower, at 90.91% and 93.64%, respectively. These results robustly demonstrate the accuracy of the proposed counting method in effectively enumerating dragon fruits at various growth stages within orchard environments.

**Figure 10:**
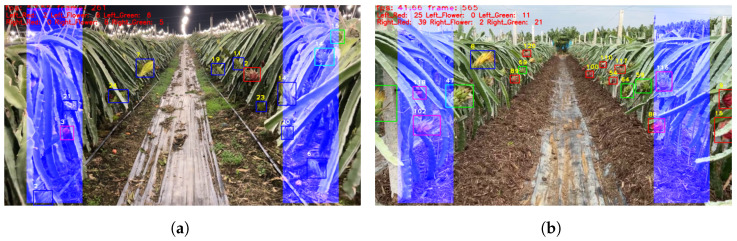
The experimental results of dragon fruit counting using the proposed method under varying lighting conditions for (**a**) night time, and (**b**) day time.

The experimental results of dragon fruit counting using the proposed method under varying lighting conditions, including night illumination from supplementary lights and direct sunlight during the day, are presented in Figure 10. Figure 10a reveals a higher abundance of dragon fruit flowers and green fruits in the considered plot, while the count of red fruits is comparatively lower. In contrast, Figure 10b shows a substantial presence of red and green fruits but a smaller count of dragon fruit flowers. Notably, Figure 10a emphasizes the challenges encountered during night time, characterized by dim lighting conditions and obstruction by branches, which contribute to the difficulty in recognizing certain dragon fruit specimens. Consequently, counting dragon fruit at different growth stages becomes more challenging during night time operations. The fluctuation in counting results displayed in Figure 10 suggests a higher propensity for misidentification and reduced counting accuracy during nocturnal activities compared to daytime operations. Conversely, Figure 10b demonstrates that direct sunlight during the day facilitates easier identification of dragon fruit, leading to improved counting outcomes. Overall, considering the results obtained from both daytime and night time counting scenarios, it can be concluded that the proposed counting method in this study exhibits robustness in diverse lighting conditions, ultimately enhancing counting efficiency.

Table 4 displays the average counting accuracies for various growth stages of dragon fruit in the test videos. The counting accuracy for dragon fruit flowers achieved a high accuracy of 97.68%, while the accuracy for counting red dragon fruit was the lowest at 91.89%, and the accuracy for green dragon fruit counting reached 93.97%. The overall average counting accuracy for all growth stages of dragon fruit was 94.51%. These results demonstrate the effectiveness of the proposed method. Additionally, the counting frequency reached 56 frames per second, indicating real-time counting capability.

### 3.3. Performance Comparison of Different Object Detection Algorithms

To assess the effectiveness of the proposed YOLOv5 model, a performance comparison was made with two other lightweight models, namely YOLOX [30] and YOLOv3-tiny [31], using the same dataset. The comparative results are presented in Table 5. Notably, YOLOv5s exhibited the smallest parameter count, while its floating-point operation (FLOPs) fell between YOLOXs and YOLOv3-tiny. Remarkably, YOLOv5s achieved the highest average detection accuracy, reaching 95%, while maintaining an impressive inference speed of just 8ms, significantly faster than YOLOXs and YOLOv3-tiny. These findings indicate that when it comes to detecting various maturity stages of dragon fruit, YOLOv5s outperforms the other models in terms of performance.

**Figure 11:**
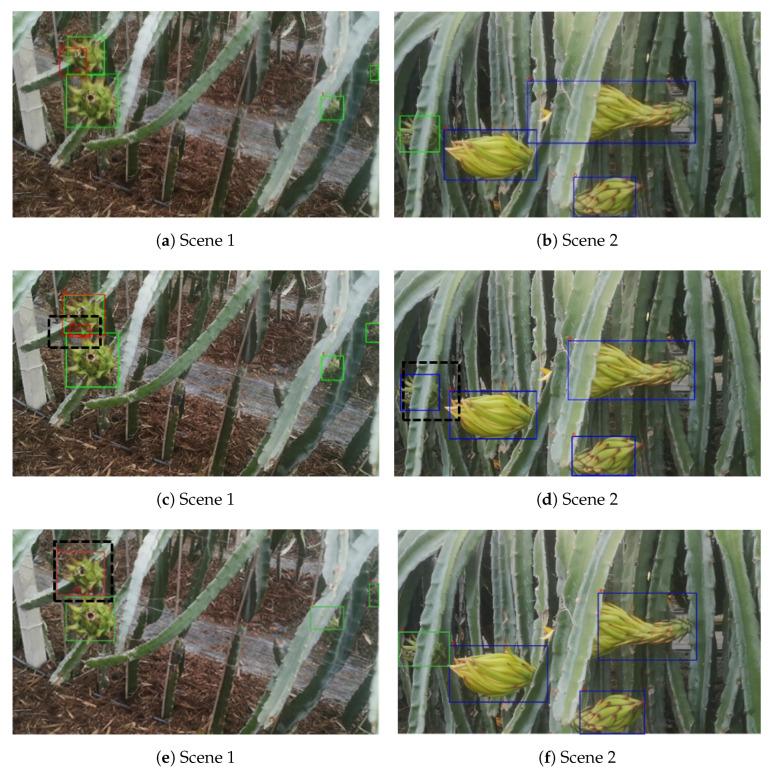
The detection performances of models in different scenes. (**a**) YOLOv5 in Scene 1, (**b**) YOLOv5 in Scene 2, (**c**) YOLOX in Scene 1, (**d**) YOLOX in Scene 2, (**e**) YOLOv3-tiny in Scene 1, and (**f**) YOLOv3-tiny in Scene 2.

Figure 11 depicts the detection performance of the three models under the same confidence level of 0.5 in two typical scenarios. Specifically, Figure 11a,b exhibits the detection results of YOLOv5, Figure 11c,d displays the results of YOLOX, and Figure 11e,f depicts the outcomes of YOLOv3-tiny. A comparative analysis of the detection results highlights that YOLOX and YOLOv3-tiny are more susceptible to false positives and false negatives when objects are occluded. For example, in Figure 11c, a single occluded red fruit target is mistakenly identified as two separate red fruit targets. In Figure 11d, an occluded green fruit target is incorrectly recognized as a dragon fruit flower. Additionally, Figure 11e shows a missed detection of a green fruit target. The experimental findings emphasize that YOLOv5 exhibits superior detection accuracy and faster detection speed compared to the other models. Moreover, YOLOv5 demonstrates enhanced robustness in occluded environments, rendering it more suitable for effectively detecting dragon fruit targets at various growth stages.

### 3.4. Performance Comparison of Different Counting Methods

**Figure 12:**
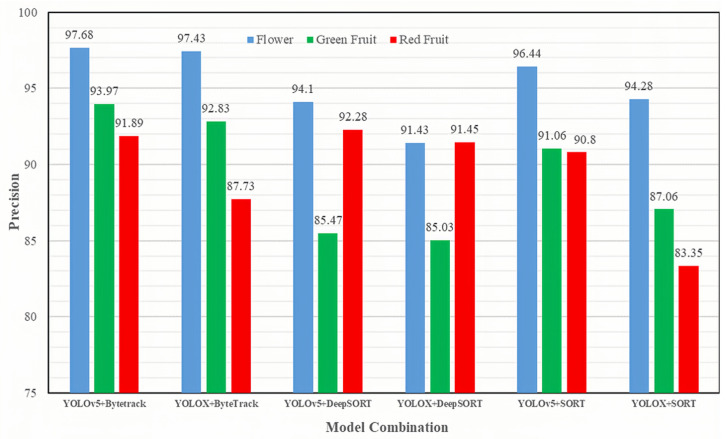
The counting accuracies achieved by different combinations of object detectors and counting methods.

To validate the counting effectiveness of the proposed method, two object detection algorithms, namely YOLOv5 and YOLOX, were employed as object detectors. These detectors were then combined with three object tracking algorithms, namely SORT, DeepSORT, and ByteTrack, resulting in six distinct combinations of counting methods. Figure 12 visually presents the counting results achieved by these six algorithms on the dragon fruit test video.

The combination of YOLOv5 and ByteTrack exhibited the highest counting accuracy of 97.68% for flower counting. For the counting results of green and red fruits, the combinations of YOLOv5 and ByteTrack, as well as YOLOv5 and DeepSORT, achieved the most favorable results, with counting accuracies of 93.97% and 92.28%, respectively. Analyzing the object detection aspect, the counting results obtained from the YOLOv5 algorithm combination generally surpassed those of the YOLOX algorithm combination. This suggests that YOLOX yielded more false detections during the detection process, consequently introducing more counting errors. Considering object tracking, ByteTrack outperformed DeepSORT and SORT when utilizing the same object detection input. The superior performance of ByteTrack can be attributed to its ability to sustain attention on detection targets with lower confidence scores, often corresponding to occluded targets. By emphasizing attention on these targets, ByteTrack facilitates tracking a higher number of targets simultaneously, ultimately leading to improved counting results.

Table 6 presents a comprehensive comparison of the six algorithm combinations in terms of counting frequency and average counting accuracy to evaluate their counting performance. The combination of YOLOv5+ByteTrack exhibited the highest counting frequency, achieving 56 frames per second. This outcome can be attributed to YOLOv5’s lower parameter count and reduced floating-point computation, resulting in faster inference speed compared to YOLOX. Additionally, ByteTrack and Sort algorithms rely solely on image correlation for target matching, eliminating the need to load the re-identification (ReID) network model. In contrast, the DeepSort algorithm requires loading the ReID network model for deep feature matching, which reduces the detection and tracking speed. In terms of average counting accuracy, the YOLOv5+ByteTrack combination also demonstrated the highest accuracy among all combinations. Considering both the counting results and counting frequency, the YOLOv5+ByteTrack combination delivered the most favorable experimental outcomes. This combination not only achieved high counting accuracy for dragon fruit at different reproductive stages but also maintained a high counting frequency.

## 4. Discussion and Future Work

The proposed counting method based on ROI regions in this paper achieves over 90% counting accuracy and a detection speed of 56 FPS, primarily focusing on high-performance GPU platforms such as the 1080 ti. However, in practical orchard applications, detection tasks often need to be executed on mobile devices. When applied to mobile devices, the speed will inevitably decrease. We have also conducted preliminary experiments on smartphones (Huawei P20), and the frame rate detection typically ranges from 18 to 24 fps, which largely meets the real-time detection requirements. Therefore, future research will aim to implement the recognition, tracking, and counting of various flowers and fruits in orchards on mobile devices, providing a more portable solution for estimating orchard yields.

In the field of agricultural detection, mobile platform-based detection has been a hot research topic. For instance, Huang [32] deployed an object detection model on edge computing devices to detect citrus images in orchards, achieving a detection accuracy of 93.32% with a processing speed of 180ms/frame. Mao [33] implemented fruit detection on CPU platforms, achieving a detection speed of 50ms/frame on smartphone platforms. This demonstrates the significant potential of fruit detection on mobile platforms.

Considering these promising developments, our future work will focus on optimizing our model and adapting it for mobile platforms. This adaptation will enable the real-time counting of fruits using these models in the field, serving as a valuable direction to meet the needs of orchard management

## 5. Conclusions

This study proposes a high-efficiency counting method for dragon fruit in orchards, which involves capturing two rows of dragon fruit plants simultaneously from the middle aisle and employing two ROI counting areas based on object detection and tracking. The proposed ROI counting method yields promising results for dragon fruit at various maturity stages. Furthermore, six combinations of two object detection algorithms and three object tracking algorithms are evaluated. Among these combinations, the YOLOv5+ByteTrack pairing achieves the most accurate counting results, exceeding 90% counting accuracy for dragon fruit flowers, green fruit, and red fruit. Notably, it also maintains a high counting frequency of 56 frames per second, ensuring efficient and rapid detection, tracking, and counting processes. The proposed counting method exhibits robustness across different environments, making it suitable for dragon fruit counting in orchards. Additionally, its applicability can be extended to quantifying other crops, providing a feasible implementation solution for fruit quantity statistics and yield prediction in orchards

## Figures and Tables

**Table 1:** Basic information of the dataset.

Training Set (5000 Images)		Validation Set (500 Images)	
**Class**	**Object Number**	**Class**	**Object Number**
Flower	13,500	Flower	1491
Green fruit	18,000	Green fruit	2073
Red fruit	20,000	Red fruit	2300
Total	51,500	Total	5864

**Table 2:** Recognition results of YOLOv5 object detection model on the test set.

Class	TP	FN	FP	TN	P%	R%	AP%	mAP%	FPS (Frame/s)
Flower	1328	163	529	3844	89.4	86.5	94.1	95.0	125
Green Fruit	1828	245	439	3352	90.5	86.6	94.8		
Red Fruit	2090	210	324	3240	92.8	89.1	96.1		

**Table 3:** The details of the test videos and their detection results using the proposed counting method.

Index	Time	Length (Frames)	Detected Flower (Ground Truth)	Detected Green Fruit (Ground Truth)	Detected Red Fruit (Ground Truth)
1	Night	2400	64 (66)	43 (53)	18 (18)
2	Night	3300	95 (95)	51 (52)	15 (18)
3	Daytime	5400	13 (13)	49 (50)	70 (77)
4	Daytime	1800	15 (16)	79 (80)	103 (110)

**Table 4:** The counting accuracies obtained by the counting method for various growth stages of dragon fruits in the test videos.

Class	${\mathrm{AP}}_{c}$%	${\mathrm{mAP}}_{c}$%	FPS (Frames/s)
Flower	97.68	94.51	56
Green Fruit	93.97		
Red Fruit	91.89		

**Table 5:** The performance comparison of object detection results with other lightweight models.

Models	Parameters (M)	Flops (G)	mAP (%)	Speed (ms/Image)
YOLOv5s	7	15.8	95.0	8
YOLOXs	8.94	17.05	93.3	12
YOLOv3-tiny	8.7	12.9	91.4	15

**Table 6:** Comparison of counting frequency and average counting accuracy using different methods.

Model Combination	${\mathrm{AP}}_{c}$%	FPS (Frame/s)
YOLOv5+ByteTrack	94.51	56
YOLOX+ByteTrack	92.66	43
YOLOv5+DeepSORT	90.61	25
YOLOX+DeepSORT	89.30	21
YOLOv5+SORT	92.76	54
YOLOX+SORT	88.23	40

## Data Availability

The data presented in this study are available upon reasonable request.
